# Iturin A Strongly Inhibits the Growth and T-2 Toxin Synthesis of *Fusarium oxysporum*: A Morphological, Cellular, and Transcriptomics Study

**DOI:** 10.3390/foods12061278

**Published:** 2023-03-17

**Authors:** Meifang Hua, Qi Deng, Mei Qiu, Yijia Deng, Lijun Sun, Zhijia Fang, Jianmeng Liao, Jian Zhao, Ravi Gooneratne

**Affiliations:** 1 Guangdong Provincial Key Laboratory of Aquatic Product Processing and Safety, Guangdong Provincial Engineering Technology Research Center of Seafood, Guangdong Province Engineering Laboratory for Marine Biological Products, Key Laboratory of Advanced Processing of Aquatic Products of Guangdong Higher Education Institution, College of Food Science and Technology, Guangdong Ocean University, Zhanjiang 525088, China; 2 College of Food Science, Southwest University, Chongqing 500715, China; 3 Zhanjiang Institute of Food and Drug Control, Zhanjiang 525022, China; 4 School of Chemical Engineering, The University of New South Wales, Sydney, NSW 2052, Australia; 5 Department of Wine, Food and Molecular Biosciences, Faculty of Agriculture and Life Sciences, Lincoln University, P.O. Box 85085, Lincoln 7657, New Zealand

**Keywords:** iturin A, *Fusarium oxysporum*, reactive oxygen species, T-2 toxin, *Tri5*

## Abstract

*Fusarium oxysporum* (*F. oxysporum*) is a common contaminant of dried fish, and the T-2 synthesis by this organism in dried fish products poses a serious public health risk. In this study, we investigated the effects of iturin A, a cyclic lipopeptide produced by *Bacillus subtilis*, on the growth and synthesis of the T-2 toxin of *F. oxysporum*, and transcriptomics was conducted. Results showed that the inhibitory effect of iturin A on *F. oxysporum* was significantly enhanced with an increase in iturin A concentrations. More specifically, compared with the control group, all indexes in the iturin A treatment group with 50 μg/mL were decreased to 24.84 mm, 0.33 × 10^6^ cfu/mL, and 5.86 ng/mL for the colony diameter, number of spores, and concentration of T-2 toxin, respectively. Furthermore, iturin A was proven to destroy the integrity of cell membranes and cause a significant increase in ROS at 25 μg/mL or 50 μg/mL. Transcriptomic analysis revealed that with the treatment of iturin A, the genes of the oxidation-reduction process were up-regulated, while the gene expression of mycelial growth, cell integrity, transmembrane transport, energy metabolism, and others were down-regulated. More importantly, the *Tri5* gene cluster was significantly inhibited. This study provided new insights into the mechanism for the inhibitory effect of iturin A on the growth and T-2 toxin synthesis of *F. oxysporum* and theoretical guidance for the application of iturin A in the preservation of dried aquatic products.

## 1. Introduction

T-2 toxin, one of the secondary metabolites of *Fusarium* sp., produced primarily by *Fusarium sporotrichioides*, is the most virulent A-type monotreme mycophenolic compound and highly UV and heat tolerant (complete degradation at 200 °C in 55 min) [1,2]. Long-term low-dose exposure to T-2 toxin can lead to DNA and RNA damage and inhibition of protein synthesis [3,4]. The T-2 toxin is cytotoxic, immunotoxic, and neurotoxic to both humans and animals [5]. The contamination rate of *Fusarium* sp. in dried fish is up to 56.7%, especially in some *Fusarium species* such as *F. oxysporum*, *Fusarium verticillium,* and *Fusarium equisetum* [6]. The temperature range of 20–30 °C and the Aw of 0.980–0.995 are optimum for T-2 toxin production [7,8]. Therefore, prevention and control of the growth and T-2 toxin synthesis through *Fusarium* sp., such as *F. oxysporum* in dried fish, are crucial to ensuring the quality and safety of these products.

Some commonly used methods to prevent mold growth in dried fish include refrigeration, irradiation, and the use of chemical and biological inhibitors. However, there are drawbacks to these methods. Specifically, refrigeration can inhibit the growth of microorganisms only for a relatively short period of time [9,10], while irradiation can result in the potential presence of benzene, toluene, and 2-alkylcyclobutanones, indicating that it will damage the human nervous system and hematopoietic system and promote the production of tumors [11]. Although chemical fungal inhibitors can effectively inhibit the growth of microorganisms, they are potentially hazardous to human health [12,13]. Hence, it is important to identify natural, non-toxic bio inhibitors. Recent studies have shown that *Bacillus subtilis* can strongly inhibit the growth and toxicity of fungi, mainly due to antimicrobial lipopeptides secreted during growth, such as surfactin, iturins, and fengycins [14,15]. Wang et al. proposed that *B. subtilis* SM21 can reduce the diameter of mycelium and activate intracellular oxidative stress, which in turn reduced the damage caused by *Rhizopus stolonifer* contamination on peaches [16]. Furthermore, *B. subtilis* B155 isolated from *Bifidobacterium subtilis* significantly inhibited the growth and spore synthesis of *Neurospora sitophila* as well as *Trichoderma* [17].

Iturins are cyclic lipopeptides composed of seven amino acid residues in a β-hydroxy fatty acid chain containing 13–19 carbon atoms. There are four iturin homologs: iturin (three types, A, C, and D), bacillopeptin, bacillomycin, and mycosubtilin. They exhibit strong antifungal activity against human pathogens and plants [18], especially against filamentous fungi including *Aspergillus carbonarius* [19], *Verticillium dahlia* [20], and *Aspergillus flavus* [21]. Iturin A exhibits the strongest ability to inhibit fungal mycelial reproduction and spore formation by destroying cell membrane integrity via interaction with cell membrane sterols and/or phospholipids [22,23]. Chen et al. found that iturins have a remarkable effect on citrus rot caused by green mold [24], and Lei et al. suggested that the minimum inhibitory concentration of iturin A against *Candida albicans* in vitro is 32 μg/mL. It has been reported that it exhibits inhibitory effects by affecting cell membrane permeability, mitochondrial potential, ROS content, and chromosomal integrity [25]. In vivo studies revealed that iturin A reduced the growth of *C. albicans* in mice without causing any toxic effects [26]. Therefore, iturin A is a promising candidate for development as a natural food preservative to inhibit fungal growth and toxin production. However, to date, there has been no study to investigate the effect of iturin A on inhibiting *Fusarium* sp. growth and toxin production.

The objective of this work was to explore the effects of iturin A on fungal growth and T-2 toxin synthesis by *F. oxysporum* in dried fish. Transcriptomics of crucial genes and pathways involved in the regulation of *F. oxysporum* growth and toxin synthesis were studied to provide molecular insight into the mechanisms of the antifungal action of this natural antifungal agent.

## 2. Materials and Methods

### 2.1. Chemicals and Experimental Strain

Iturin A was purchased from Sigma-Aldrich (America); the T-2 toxin standard from Enzo Life Science (Farmingdale, New York, USA). Wild-type *F. oxysporum* (GDMCC 60825), which has a strong capacity to synthesize T-2 toxin, was isolated from dried fish.

### 2.2. Colony Growth Analysis

A potato dextrose agar (PDA, Beijing, China) medium with different concentrations of iturin A (0, 25, 50, 100 μg/mL) was prepared. The 5 mm mycelium disk of *F. oxysporum* was inoculated into PDA medium and cultured for 7 days at 28 °C. The colony morphology was photographed.

### 2.3. Sporulation Capacity Analysis

Carboxymethyl cellulose (CMC, Yantai, China) medium with different concentrations of iturin A (0, 25, 50, 100 μg/mL) was prepared. The *F. oxysporum* was inoculated into the CMC medium, followed by incubation in a shaking incubator at 120 rpm/min at 28 °C for 7 days. The number of fungal spores was calculated using a CX23 optical microscope (Puch, Shanghai, China).

### 2.4. Analysis of T-2 Toxin Synthesis

T-2 toxin synthesis in glucose yeast medium (GYM) and the determination of T-2 toxin were performed on the basis of the method described by Qiu et al. [27]. *F. oxysporum* was inoculated into the GYM with a range of various concentrations of iturin A (0, 25, 50, 100 μg/mL) and incubated at 28 °C for 15 days; after that, the concentration of T-2 toxin was determined by LC-MS/MS.

T-2 toxin analysis was carried out on the Thermo Scientific Surveyor HPLC system, which is made up of an online degasser, a Surveyor MS Pump Plus, and a Thermo TSQ Quantum Access tandem mass spectrometer equipped with an electrospray ionization (ESI) source and a Surveyor Autosampler Plus (Waltham, MA, USA). Hypersil GOLD column (5 μm, 100 mm × 2.1 mm) (Thermo Scientific, Waltham, MA, USA) was used for separation at a flow rate of 0.25 mL/min at 35 °C. The mobile phase, including methanol (A) and water containing 5 mM ammonium acetate and 0.1% formic acid (B). The gradient elution procedure was as follows: 0 min 30% A, 3.0 min 90% A, 5 min 90% A, 5.1 min to 8 min 30% A. MS/MS was performed on both a positive (ESI+) mode electrospray ionization source and a triple quad mass spectrometer (Shimazu, Kyoto, Japan). The ionization source parameters were set as follows: capillary temperature: 350 °C; spray voltage: 4500 V; tube lens bias: 118 V; sheath gas pressure: 35 au; collision energy: 1.5 eV; ion scavenging pressure: 0 au; skimming offset: 0; collision pressure: 1.5 mTorr; auxiliary gas pressure: 15 au.

### 2.5. Scanning Electron Microscope (SEM) Analysis

Mycelial and spore samples from the culture plates used for the mycelial growth analyses (see Section 2.2) were used for scanning electron microscope analysis. The morphology of mycelial and spore cells was examined using a JSM-7610F SEM (JEOL Ltd., Tokyo, Japan).

### 2.6. Membrane Permeability Analysis

Membrane samples with permeability were obtained from the culture plates (see Section 2.2). The hyphae cells were washed 3 times with 0.01 M phosphate buffer (pH 7.4). Next, the hyphae were added to a PI staining solution (Sangon Bioengineering Co., Ltd., Shanghai, China), and cultivated at 28 °C for 30 min to observe the hyphae color.

### 2.7. Analysis of Reactive Oxygen Species

Reactive oxygen samples were prepared from the culture medium used for T-2 toxin synthesis (see Section 2.5). The hyphae cells were washed 3 times with 0.01 M phosphate buffer (pH 7.4). Next, the hyphae were added to a DCFH-DA (2,7-Dichlorodihydrofluorescein diacetate) (Nanjing Jiancheng Bioengineering Institute, Nanjing, China) and incubated at 28 °C for 30 min to observe the hyphae color. In addition, the determination of ROS concentration in mycelium was performed according to the method of Yu et al. [28] with minor modifications. The concentration of ROS was measured under the following conditions: slit width: 5 nm, excitation wavelength: 500 nm, and emission wavelength range: 525 nm via a RF-5301PC fluorescence spectrophotometer (Shimadzu Co., Kyoto, Japan). All data were acquired repeatedly for three times.

### 2.8. Transcriptome Analysis

#### 2.8.1. Isolation of Total RNA, cDNA Library Construction, and Sequencing

The samples for transcriptomic analysis were obtained from the culture medium for T-2 toxin synthesis (see Section 2.3). Total ribonucleic acid (RNA) was obtained from each sample using a TRIzolTMLS Reagent Kit (Invitrogen, Carlsbad, CA, USA). Each experiment was performed three times. A nanophotometer spectrophotometer (IMPLEN, Germany) was used to determine ribonucleic acid purity with the OD260/280 value of 1.8–2.2 and the OD260/230 value of >2.0, and a Bioanalyzer 2100 system (America-Agilent) assay kit was used to accurately assess RNA integrity with a RIN value of >7.0 followed by identification with denaturing agarose gel electrophoresis.

The mRNA with polyA tails was enriched by Oligo(dT) magnetic beads and subsequently randomly interrupted with divalent cations in the NEB fragmentation buffer. The mRNA fragment was selected as the template, and the first strand of cDNA in the M-MuLV reverse transcriptase system was constructed by the random oligonucleotides which were treated as primers, followed by retrogradation of the RNA strand with RNaseH and synthesis of the second strand of cDNA with dNTPs using the DNA polymerase I system. The insertion length of 150 bp in the library was obtained by using an Agilent 2100 bioanalyzer, and the efficient concentration of the library was quantified by qRT-PCR (>2 nM). The target downstream data volumes were sequenced on an Illumina Novaseq™6000 (LC-Bio Technology Co., Ltd., Hangzhou, China) on the grounds of the manufacturer’s instructions.

#### 2.8.2. Data Analysis, Differentially Expressed Genes Screening, and Bioinformatics Analysis

Each sample transcriptomic library obtained by sequencing was transformed into raw sequencing sequence data and analyzed for Consensus Assessment of Sequence and Variation (CASAVA) base sequence identification. The validated data were filtered for RNA sequences by the software HISAT2 V2.0.5, compared with the reference genome of *F. oxysporum* downloaded from NCBI (https://www.ncbi.nlm.nih.gov/g (accessed on 22 January 2021)). The data generated by sequencing were processed using the software HTseq (version: 0.12.5). Efficient and accurate mapping of these reads to genes enables comparability of estimated gene expression levels across genes and across experiments. The differentially expressed genes were analyzed and compared using DESeq 2R software (Versions 3.16). The Benjamini and Hochberg method was employed to regulate the *p*-values, and the parameters of *p* < 0.05 and ∣Log_2_ Fold change∣ ≥ 2 were set as differentially expressed genes (DEGs).

### 2.9. Quantitative Real-Time PCR Analysis

The genes related to the growth and toxin synthesis of *F. oxysporum* were selected for transcriptional validation. The translation elongation factor 1 alpha (FoEF1a) gene was used as an internal control based on the transcriptional sequence of *F. oxysporum* genes. The relative transcript levels were detected using the CFX96 real-time fluorescence quantitative PCR system, CFX96 Touch Real-Time PCR (BIO-RAD, Hercules, CA, USA) (Table 1). Primer Premier 5.0 was used to design the primers, and it was synthesized by Sangon Biotechnology (Shanghai, China). The relative transcript levels of the tested genes were calculated using the 2^−(∆∆Ct)^ method.

### 2.10. Statistical Analysis

The mean ± standard deviation (SD) was represented as the expression of results (*n* = 3 for each group). A one-way analysis of variance (ANOVA) followed by Tukey’s test was performed by the IBM SPSS Statistics 26.0 software (SPSS Inc., Chicago, IL, USA). A *p*-value < 0.05 was considered significant. The results of colony size, number of spores, and T-2 toxin synthesis were analyzed by one-way analysis of variance (ANOVA) to observe the effects of iturin A on the growth and metabolism of *F. oxysporum*.

## 3. Results

### 3.1. Effects of Iturin A on Colony Diameter

**Figure 1:**
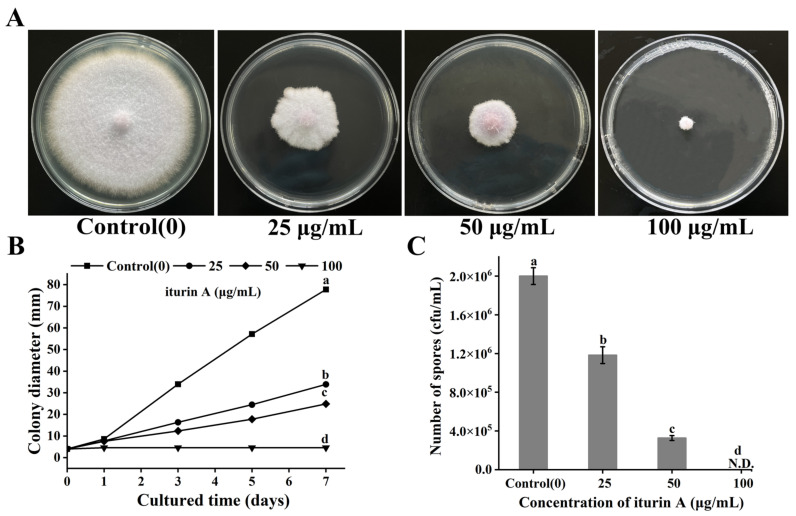
(**A**) Colony morphology of *F. oxysporum* in PDA medium cultured with iturin A for 7 days; (**B**) effects of iturin A on colony diameter of *F. oxysporum*; (**C**) effects of iturin A on *F. oxysporum* spore production (different letters represent significant differences between treatments, *p* < 0.001; N.D.: Not Detection).

The effect of iturin A (0–100 μg/mL) treatment on the growth of *F. oxysporum* is shown in Figure 1. In the control group, the colony diameter of *F. oxysporum* was 77.75 mm after 7 days of culture. Iturin A (0, 25, 50, and 100 μg/mL) inhibited the growth and reduced the colony diameter of the organism significantly (Figure 1A), and the effect was dose-dependent (*p* < 0.001) (Figure 1B). Similarly, the inhibitory effect of iturin A on *F. oxysporum* spore formation was also dose-dependent. The spore count in the control group was 2.0 × 10^6^ cfu/mL, but the number of spores in the 25 and 50 μg/mL iturin A groups was significantly smaller, with values of 1.18 × 10^6^ cfu/mL and 3.27 × 10^5^ cfu/mL (*p* < 0.001), respectively. At the concentration of 100 μg/mL, the hyphal growth and sporulation of *F. oxysporum* were completely inhibited (Figure 1C). Previous studies have shown that iturin A can be used for biological control of crop pathogens, such as mycelium growth and conidial synthesis in rice pestilentum and gray mold in wheat [29,30]. In addition, iturin A can also be used to prevent postharvest contamination of certain fungal pathogens, whose inhibition rates against *Penicillium digitatum* and *Botrytis cinerea* on lemon and strawberry surfaces were 68.6% and 74.1%, respectively [31,32]. This research showed that iturin A at 25 μg/mL and 50 μg/mL resulted in a 50.35% and 66.58% decrease in mycelial growth and a 50.83% and 83.67% inhibition of spore formation, indicating that iturin A could be used to effectively prevent and control the contamination of *F. oxysporum* in aquatic dry products.

### 3.2. Effects of Iturin A on T-2 Toxin Synthesis

**Figure 2:**
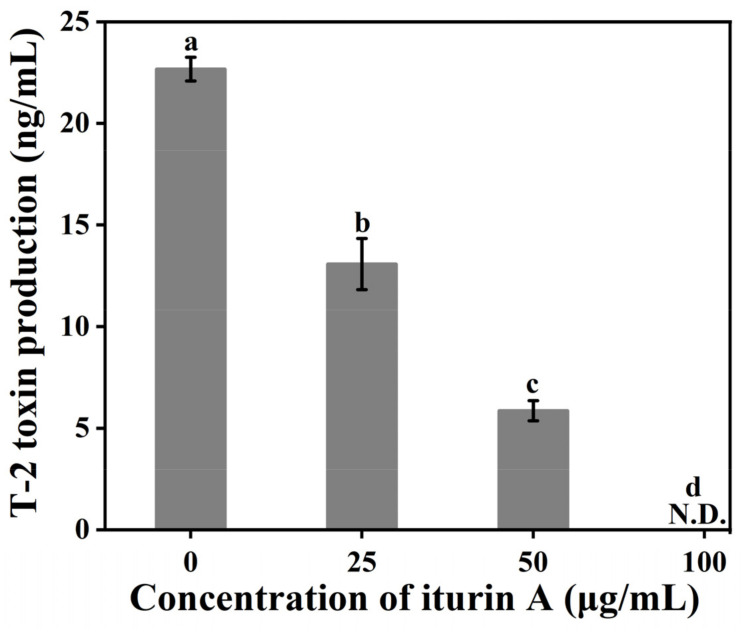
Effects of iturin A on T-2 toxin synthesis by *F. oxysporum*. (Different letters represent significant differences, *p* < 0.001; N.D.: Not Detection).

Iturin A significantly inhibited the synthesis of T-2 toxin by *F. oxysporum,* and the effect was also dose-dependent (Figure 2). The T-2 toxin concentration in the control group was 22.67 ng/mL, while at 25 and 50 μg/mL iturin A, it was 13.08 ng/mL and 5.86 ng/mL, representing an inhibition rate of 42.30% and 74.15%, respectively. These results are in accordance with the foregone publication that iturin A can significantly inhibit ochratoxin A (OTA) produced via *Aspergillus carbonarius* and aflatoxin B_1_ (AFB_1_) production by *Aspergillus flavus* [33], confirming the effect of iturin A as an effective inhibitor of mycotoxin synthesis by different fungal species.

### 3.3. Effects of Iturin A on the Morphology of Hyphae and Spores

**Figure 3:**
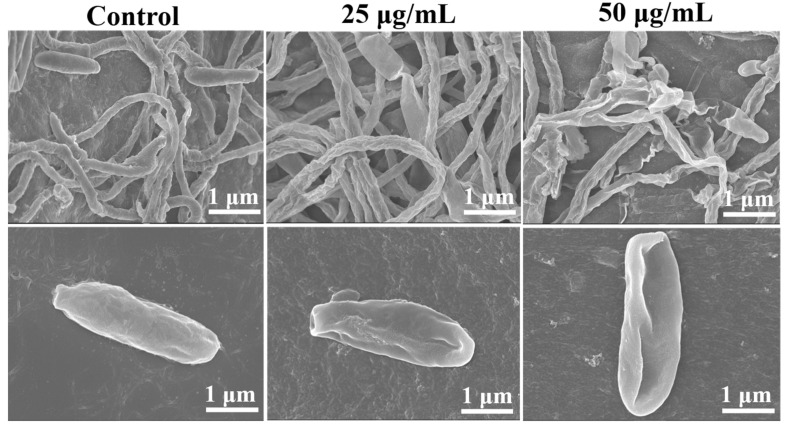
Effects of 25 and 50 μg/mL iturin A on hyphal and spore morphology of *F. oxysporum* revealed by scanning electron microscopy.

As shown in Figure 3, in the control, the hyphae and spores were fully formed, round, and translucent. Following treatment with 25 μg/mL iturin A, the hyphae and spores of *F. oxysporum* were damaged, showing unevenness, shrinkage, and folds. At 50 μg/mL iturin A, the effects were more pronounced, showing severely deformed hyphal and spore structures, shriveling, and shrinkage. The results are consistent with the studies of Han et al. [34], showing that iturin A can inhibit the growth of *F. oxysporum* with consequent drastic changes in cell morphology and structure, including cell surface unevenness, cytoplasm shrinkage, and damaged organelles. Similar results have been reported with iturin A on *Verticillium dahliae* and *Aspergillus carbonarius* [20].

### 3.4. Effects of Iturin A on Cell Membrane Permeability

**Figure 4:**
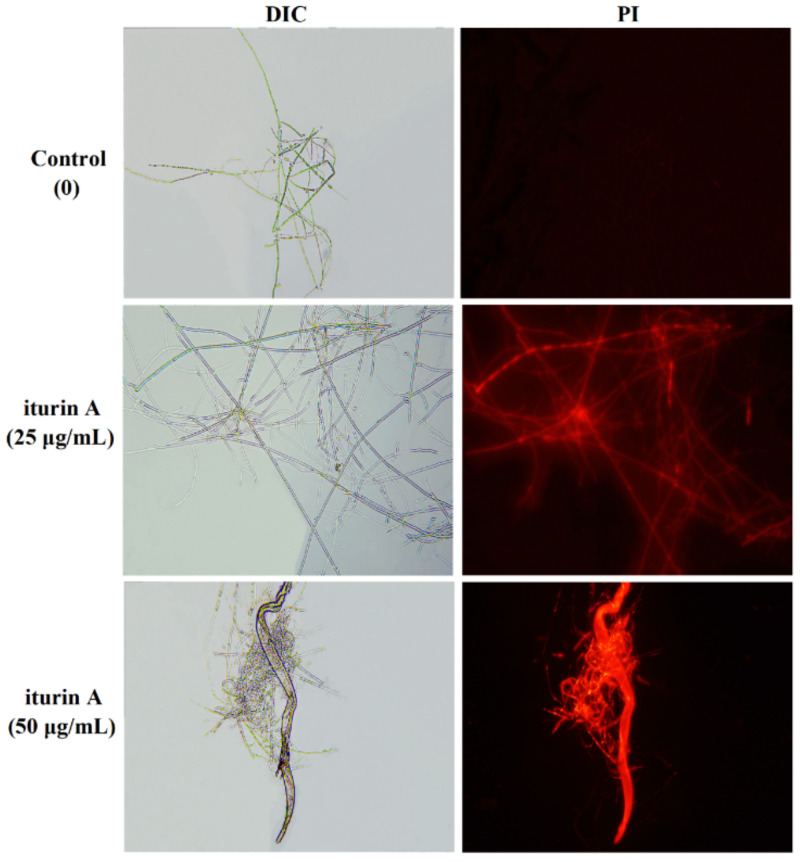
Effects of 25 and 50 μg/mL iturin A on the cell membrane permeability of *F. oxysporum* as examined by optical microscopy.

The cell membrane is a semi-elastic, semipermeable structure with phospholipids as one of the main components, which is responsible for maintaining the structural integrity of cells and protecting intracellular components [35]. PI staining was used to examine the effect of iturin A on the cell membrane integrity of *F. oxysporum.* No fluorescence of hyphae was observed in the control group, while in samples treated with 25 and 50 μg/mL iturin A, the hyphae appeared red and the color depth was dose-dependent (Figure 4). These results indicate that the structural integrity of *F. oxysporum* was damaged significantly by the iturin A treatments.

### 3.5. Effects of Iturin A on Reactive Oxygen Species

**Figure 5:**
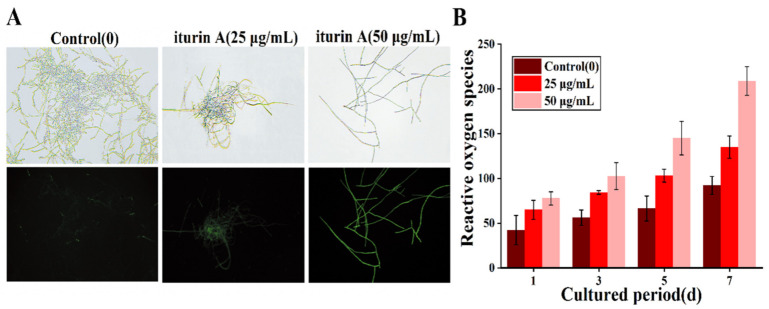
Effects of 25 and 50 μg/mL iturin A on intracellular reactive oxygen species (ROS) in *F. oxysporum* as examined by biofluorescence microscopy (**A**) and fluorescence spectrophotometry (**B**).

Reactive oxygen species (ROS) are significant to many aspects of fungal life, such as mycelial growth and toxin synthesis [36,37]. Compared with the control, the *F. oxysporum* hyphae in the treatment group exhibited obvious green fluorescence following treatment with 25 and 50 μg/mL iturin A, indicating that iturin A caused a ROS imbalance (Figure 5A). Strong green fluorescence was observed in the treated group, with fluorescence in the control group being rather weak. In addition, ROS concentration in *F. oxysporum* was observed to increase during the 7-day incubation period for all samples. However, the increase was significantly greater with iturin A-treated samples, and the effects were positively correlated with iturin A concentration. (Figure 5B). This is consistent with the findings of Lei et al. [25], who reported an accumulation of ROS in iturin-treated *Candida albicans* cells by DCFH-DA. Thus, iturin A caused an increase in the reactive oxygen concentration in *F. oxysporum*, which could lead to oxidative stress in the organism and affect its growth and T-2 toxin synthesis.

### 3.6. Analysis of GO Function and KEGG Pathway of DEGs

**Figure 6:**
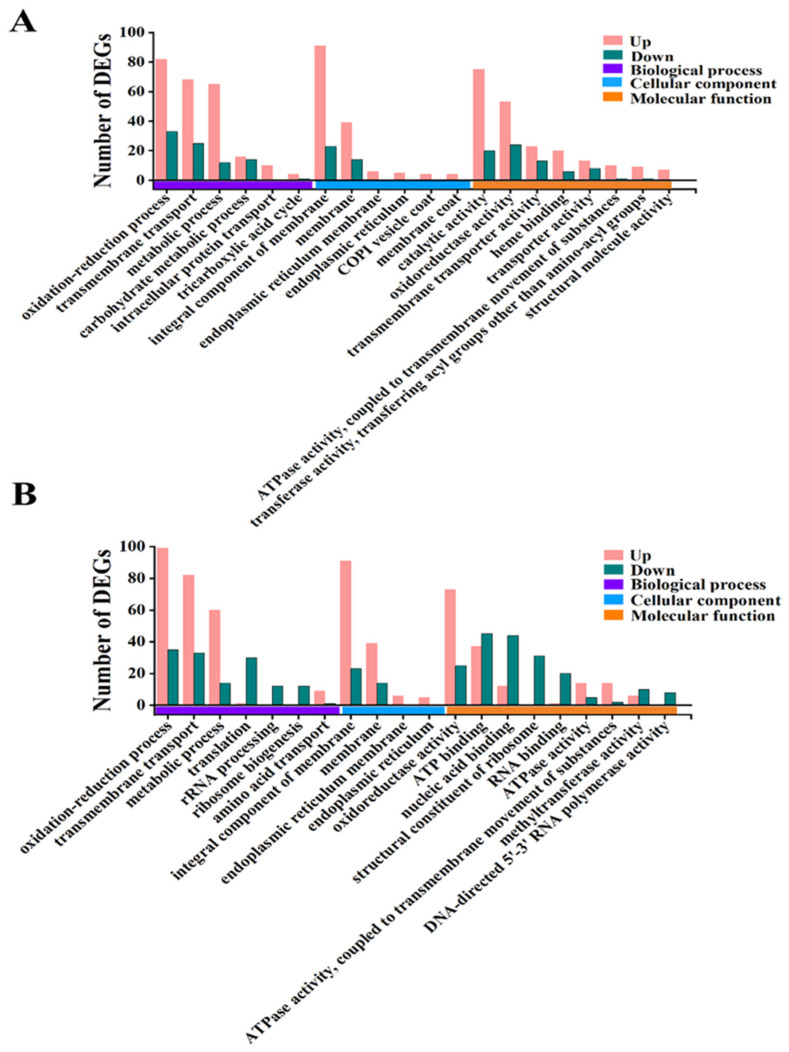
GO functional analysis of differential genes in *F. oxysporum* treated with 25 (**A**) and 50 μg/mL (**B**) iturin A.

To further define the functions, metabolic pathways, and interrelationships of DEGs, bioinformatics techniques including Kyoto Encyclopedia of Genes and Genomes (KEGG) pathway enrichment analysis and Gene Ontology (GO) enrichment were performed. The GO analysis (Figure 6) demonstrated that the top 15 functional annotations of DEGs were significantly up-/down-regulated in the iturin A-treated groups at both 25 and 50 μg/mL doses compared with the control group. Compared with the control group, DEGs in the 25 and 50 μg/mL iturin A treatment groups were enriched mainly in the oxidation-reduction process, transmembrane transport, the metabolic process of biological processes domain, an integral component of membrane and membrane in the cellular component domain, and catalytic activity and oxidoreductase activity in the molecular function domain.

**Figure 7:**
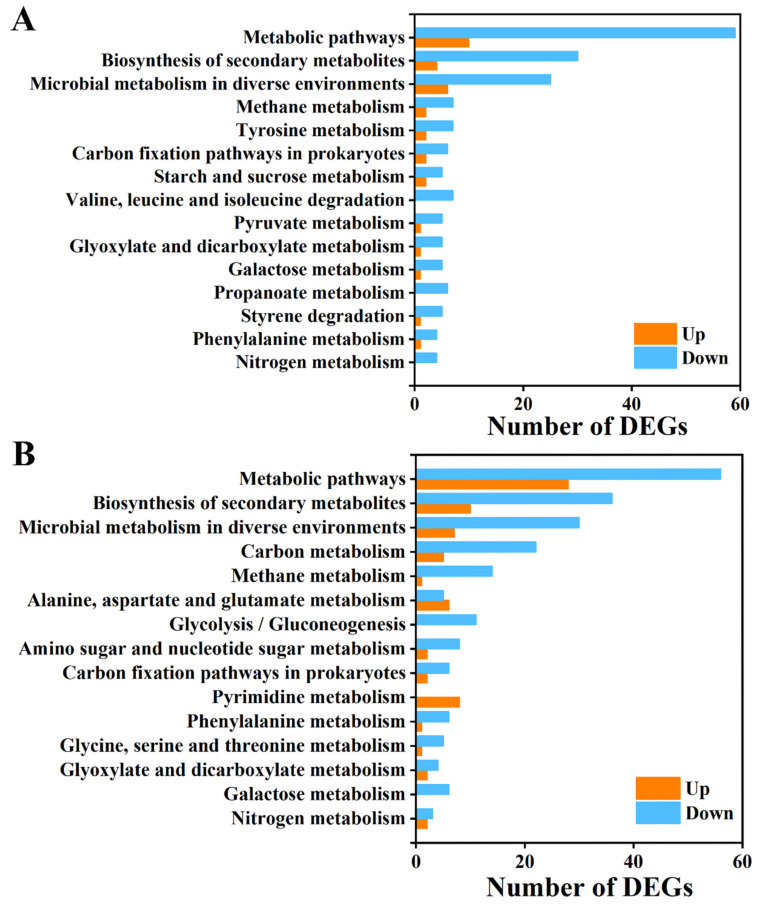
KEGG signal pathway enrichment analysis of differential genes in *F. oxysporum* treated with 25 (**A**) and 50 (**B**) μg/mL iturin A.

The DEGs of the 25 and 50 μg/mL iturin A treatment groups were subjected to KEGG pathway enrichment analysis (Figure 7). The DEGs are significantly enriched in metabolic pathways, biosynthesis of secondary metabolites, and microbial metabolism in different environments.

**Figure 8:**
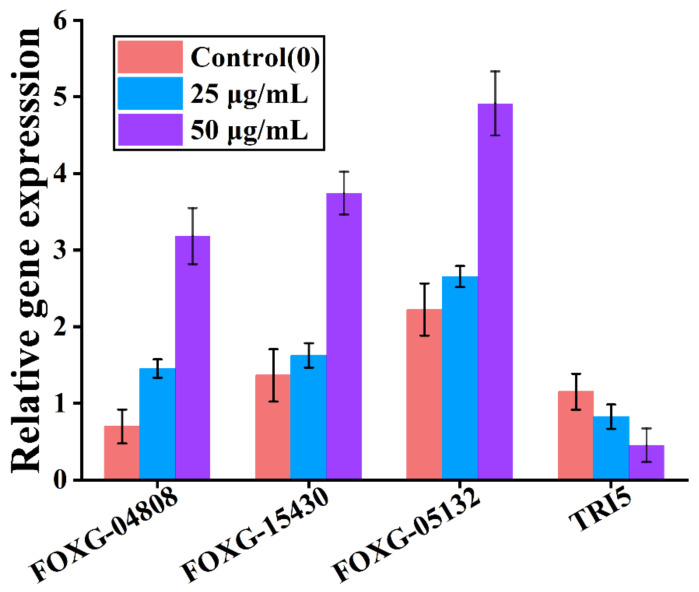
Quantitative reverse transcription PCR verification of the differential genes in *F. oxysporum* following treatment with 25 and 50 μg/mL iturin A.

qRT-PCR confirmed the expression of the DEGs identified by the transcriptome analysis (Figure 8), indicating that the transcriptome analysis is reliable.

### 3.7. Functional Analysis of DEG

Based on the data from the current study, some DEGs are considered major genes related to mycelial growth, T-2 toxin biosynthesis, cell integrity, transmembrane transport, redox, and energy metabolism.

#### 3.7.1. Mycelial Growth

The expression of genes related to hyphal growth was down-regulated following iturin A treatment of *F. oxysporum*. The down-regulated genes in the 25 μg/mL iturin A group (Table 2) included the DNA repair protein rhp51 (FOXG_00235) and myb-like DNA-binding protein FlbD (FOXG_05220). Some down-regulated genes in the 50 μg/mL iturin A group (Table 3) were similar to those in the 25 μg/mL iturin A group. In addition, encoding tRNA (FOXG_19811) and ribosome proteins (FOXG_02005, FOXG_02122, and FOXG_05075) were also significantly down-regulated. Ribosome proteins and tRNA are important parts of the translation and transport of cellular ribosomes, all of which are closely related to fungal growth. Therefore, both 25 and 50 μg/mL iturin A treatments inhibited *F. oxysporum* growth, and the effect was dose-dependent.

#### 3.7.2. T-2 Toxin Synthesis

T-2 synthesis is regulated by multiple trichothecene synthetic gene clusters, such as *Tri3*, *Tri4*, *Tri5*, *Tri7*, *Tri8*, *Tri11*, and *Tri13* [38,39]. *Tri5* is an initiator of trichothecene toxin synthesis and plays an important role in T-2 toxin synthesis [40]. A significant down-regulation of the expression of the gene encoding *Tri5* was observed following exposure of *F. oxysporum* to iturin A (Table 2 and Table 3), which was consistent with T-2 toxin synthesis. Jiang et al. proposed that iturin A can inhibit T-2 toxin biosynthesis by down-regulating the expression levels of the enzymes encoding cytochrome P450 and halogenase [23]. This result indicates iturin A inhibited T-2 toxin biosynthesis by significantly down-regulating the expression of *Tri5*, a gene of the T-2 toxin biosynthesis cluster.

#### 3.7.3. Cell Integrity

Fungal cell walls maintain cell morphology and resist environmental damage, thus serving as potential targets for antifungal therapy [41]. Cellulose, glucan, and chitin are the key components of the cell wall and are responsible for the structural integrity of the cell wall [42]. Iturin A at 25 μg/mL significantly affected carbohydrate metabolism related to cell wall biosynthesis, such as glucose metabolism, starch metabolism, and amino acid metabolism. Among them, the expression of genes encoding endoglucanase, beta-glucosidase, glucoamylase, alpha-amylase, and other functional enzymes responsible for cell wall structure were down-regulated, indicating that the integrity of the fungal cell wall was impaired following iturin A treatment. The expression of the chitin-encoding gene (FOXG_19879) was significantly up-regulated in the 50 μg/mL iturin A treatment group (Table 3). Jiang et al. reported that the cell wall structure was destroyed following iturin A treatment of carbapenem cells, resulting in more demand for chitin to generate the cell wall [23].

Cell membranes are a bilayer of phospholipids and proteins interspersed with the cell structure to adjust the transport of materials into and within the cell. Iturin A treatment at 25 and 50 μg/mL, the differential genes of *F. oxysporum* were mainly enriched in integral components of the membrane with more up-regulated genes than down-regulated genes. In addition, the endoplasmic reticulum membrane, endoplasmic reticulum (endoplasmic reticulum), COPI vesicle coat, and outer membrane coat were also up-regulated. These results indicated that iturin A increased membrane permeability but did not completely eliminate membrane composition. Hence, the up-regulated genes were only a passive emergency measure.

Ergosterol is the main sterol component of the cell membranes of fungi, and it performs essential regulating functions on cell structure, proliferation, development, and infiltration [43]. The steroid metabolism process of *F. oxysporum* was significantly up-regulated following iturin A treatment. Transcription levels of genes encoding ergosterol were up-regulated following 25 and 50 μg/mL iturin A treatment, including sterol 25-C-methyltransferase (FOXG_08765), sterol 15-demethylase (FOXG_11555), sterol carrier protein 2 (FOXG_12787), delta25(25(1))-sterol reductase (FOXG_05355), and C-5 sterol desaturase (FOXG_10530) was also up-regulated. Sterol 15-demethylase, as one of the cytochrome P-550 (cytochrome P550) protein superfamily, can catalyze the main steps of ergosterol biosynthesis and has high substrate specificity [44]. Delta 25 (25 (1))-sterol reductase is a key element in the aerobic metabolism of *Saccharomyces cerevisiae* [45]. Taken together, iturin A has a high affinity for ergosterol and significantly affects fungal cell wall and membrane synthesis and composition [46]. Hence, ergosterol acted as a crucial target for the iturin A antifungal action.

#### 3.7.4. Transmembrane Transport

The cell membrane is a barrier for the transport of substances inside and outside the cell, and the transmembrane transport of water-soluble molecules depends on transporters. On exposure to 25 and 50 μg/mL iturin A, the gene expression of the code MFS transporter, the AGZA family, xanthine/uracil permease (FOXG_15621), and the ABC transporter (FOXG_01535) were significantly downregulated. The ABC transporter is an external transport pump that has the function of effluxing drugs, can relieve the stress of antibiotics, and is an important member of fungal drug resistance. Hence, treatment with 50 μg/mL iturin A of *F. oxysporum* reduced the efflux ability of *F. oxysporum*, making it more vulnerable to iturin A. Encoding MFS transporter, SP family, general alpha glucoside: H^+^ symporter (FOXG_05866, FOXG_10778, FOXG_13625) were significantly up-regulated in the 25 and 50 μg/mL iturin A group. The MFS transporter, as a secondary active transporter, is responsible for transporting sugars and glucosides into cells. Hence, treatment with 50 μg/mL iturin A of *F. oxysporum* reduced the efflux ability of *F. oxysporum*, making it more vulnerable to iturin A (Table 3). On the contrary, in order to sustain cell growth and normal material transport, the expression of some energy-related genes needs to be up-regulated.

#### 3.7.5. Redox

The redox balance in cells can be affected by changes in environmental conditions. Fungi are capable of responding to oxidative stress by provoking non-enzymatic and enzymatic defense systems to prevent cellular dysfunction and even cell death [47]. Iturin A induced the accumulation of intracellular ROS (Figure 5) in *F. oxysporum*, which is a hallmark of oxidative stress. The expression of genes encoding catalase (FOXG_05808), peroxidase (FOXG_15530), oxidoreductase (FOXG_05132), catalase-peroxidase (FOXG_12260), and glutathione S-transferase (FOXG_07591 and FOXG_13656) was up-regulated following 25 and 50 μg/mL iturin A treatment of *F. oxysporum*. Glutathione is the central and foremost non-enzymatic system that protects cells from oxidative stress. Glutathione S-transferase belongs to the system of glutathione, which results in the combination of glutathione with multifarious exogenous compounds to decrease its toxicity by catalyzing processes [48]. In addition, 50 μg/mL iturin A treated *F. oxysporum*, encoding oxidoreductase (FOXG_15750, FOXG_05132, FOXG_00572, FOXG_09098, FOXG_07588, FOXG_05703), superoxide dismutase (FOXG_03076) and S-(hydroxymethyl) glutathione dehydrogenase (FOXG_10652) gene expression was significantly up-regulated. Hence, with the increase in iturin A concentration, the number and expression of genes encoding antioxidants increased (the detailed information is shown in Table 2 and Table 3).

#### 3.7.6. Energy Metabolism

Mitochondria are considered the main energy production center of eukaryotes and the site of aerobic respiration, where the enzymes related to energy metabolism in mitochondria can regulate cellular respiration and energy metabolism [49]. Iturin A at both 25 and 50 μg/mL on *F. oxysporum* resulted in the genes encoding mannitol-1-phosphate dehydrogenase (FOXG_07519) and acid phosphatase (FOXG_12362, FOXG_07382, and FOXG_10756) being down-regulated in the pentose phosphate pathway. Qi et al. proposed that apoptosis in fungal cells can be induced by lipopeptides through a mitochondria-dependent pathway based on phosphatidylserine externalization, cytochrome c release, ROS accumulation, and caspase-like activity upon lipopeptide treatment [50]. Up-regulation of genes encoding or 3-isopropylmalate dehydrogenase (FOXG_05798), ketol-acid reductoisomerase (FOXG_05288) gene in the 25 μg/mL iturin A group, and 6-phosphofructokinase (FOXG_06150), hexokinase (FOXG_12010, FOXG_03031), enolase (FOXG_00035), and triosephosphate isomerase (FOXG_02233) genes in the 50 μg/mL iturin A group means that tricarboxylic acid metabolism in *F. oxysporum* was accelerated and more energy was generated to adapt the body to the stress of iturin A. Meanwhile, in the 50 μg/mL iturin A group, encoded 6-phosphofructokinase (FOXG_06150), hexokinase (FOXG_12010, FOXG_03031), enolase (FOXG_00035), and triosephosphate isomerase (FOXG_02233) were significantly up-regulated. The glycolytic pathway, which utilizes enzymes such as 6-phosphofructokinase, hexokinase, and enolase, is important to release energy from glucose. The ATP/AMP ratio is of great significance in the regulation of 6-phosphofructokinase-1 activity. When the cellular ATP concentration is high, 6-phosphofructokinase is almost inactive, and glycolysis is low. In contrast, during high energy consumption, the ratio of ATP/AMP in the cell decreases, the enzyme activity increases, and the decomposition of sugar is accelerated to supply energy. In addition, the genes encoding acyl-CoA dehydrogenase (FOXG_07887, FOXG_05118), acetyl-coenzyme A synthetase (FOXG_00735), and acetyl-CoA acyltransferase (FOXG_11385, FOXG_06200, FOXG_08599) were significantly up-regulated. Hence, when *F. oxysporum* is exposed to iturin A, glycolysis is accelerated to generate energy for cells via the glycolytic pathway and TCA cycle.

Notably, the gene encoding endonuclease G (FOXG_02255) was not expressed in the control group but was highly expressed in the 25 and 50 μg/mL iturin A groups. Endonuclease G is a mitochondria-specific nuclease that transforms *F. oxysporum* chromatin DNA into nucleosomal fragments independent of caspases. Therefore, overexpression of endonuclease G observed in the mitochondria of *F. oxysporum* can induce caspase-independent apoptosis under the stress of iturin A exposure.

## 4. Conclusions

Iturin A exhibited a strong inhibitory effect on both mycelial growth and T-2 biosynthesis in *F. oxysporum*. The treatment of iturin A at 100 ug/mL completely prevented mycelial growth. Amounts of 25 μg/mL iturin A and 50 μg/mL inhibited mycelial growth by 50.35% and 66.58% and T-2 synthesis by 42.30% and 74.15%, respectively. Iturin A not only affected the integrity of fungal cell membranes but also induced a stress response and a significant increase in ROS in the fungal cells. All effects were consistent with observed changes in the expression of relevant genes. Specifically, *Tri5*, a biosynthetic cluster gene of the T-2 toxin, was markedly down-regulated. This study shows that iturin A, a bioactive compound that is green, safe, and efficient, can be used in the preparation of different food-grade biopolymer matrices to improve the bioefficacy against fungi and mycotoxins. We believe that iturin A can serve as a potential anti-mycotoxin substitute for chemical anti-fungal agents to improve food quality and safety.

## Figures and Tables

**Table 1:** A sequence of gene primers for RT-PCR.

Gene	Primer Extension Direction	Primer Sequence
FoEF1α	Forward	CGGTACTGGTGAGTTCGAGGCT
	Reverse	TGTTGATGGCGACAATGAGGTT
FOXG-05808	Forward Reverse	AACAAGACTCCCGAGGATAAGG CATCATCAGGGCCAGCTACAT
FOXG-15530	Forward Reverse	ACCGCATCACCACCCCTA TCGGAGGTAAGGCTTGACG
FOXG-05132	Forward Reverse	ACTGCATCGATCTGCTCTACCT CGCCGTGAAGTTGCTGATA
*Tri5*	Forward Reverse	CTTTCCCACCGAGTATTTTCT AATCTCATGGAGGCGGTATCG

**Table 2:** Differentially expressed genes in *F. oxysporum* exposed to 25 μg/mL iturin A compared with the control.

Gene ID	log_2_ (IA/CK)	*p*-Value	Annotated Gene Function	Gene ID	log_2_ (IA/CK)	*p*-Value	Annotated Gene Function
DOWN-REGULATED							
FOXG-00235	−1.551	0.027	DNA repair protein rhp51	FOXG-05866	−1.978	0.001	MFS transporter, SP family, general alpha glucoside: H^+^ symporter
FOXG-05220	−1.198	0.037	myb-like DNA-binding protein FlbD	FOXG-05595	−2.103	0.005	cytochrome-b5 reductase
Tri5	−1.521	0.256	synthetic enzyme gene	FOXG-05595	−2.052	0.006	cytochrome P550 oxidoreductase
FOXG-05120	−1.311	0.0322	endoglucanase	FOXG-06551	−1.957	0.002	choline dehydrogenase
FOXG-15550	−3.83	0	beta-glucosidase	FOXG-16238	−1.677	0.005	alcohol dehydrogenase
FOXG-13566	−2.392	0	glucoamylase	FOXG-07519	−1.581	0.002	mannitol-1-phosphate dehydrogenase
FOXG-16920	−1.205	0.037	alpha-amylase	FOXG-12362	−1.108	0.053	acid phosphatase
FOXG-15621	−5.085	0.015	MFS transporter, AGZA family, xanthine/uracil permease				
UP-REGULATED							
FOXG-08765	3.065	0.018	sterol 25-C-methyltransferase	FOXG-01307	2.928	0.01	glutathione S-transferase
FOXG-12787	2.756	0.0553	sterol carrier protein 2	FOXG-07591	2.658	0.01	glutathione S-transferase
FOXG-11555	2.561	0.015	cytochrome P450, family 51 (sterol 15-demethylase)	FOXG-03656	1.557	0.052	glutathione S-transferase
FOXG-05355	1.352	0.375	delta25(25(1))-sterol reductasedelta	FOXG-05798	2.755	0.168	3-isopropylmalate dehydrogenase
FOXG-10778	2.6	0.088	MFS transporter, SP family, general alpha glucoside: H^+^ symporter	FOXG-05288	1.556	0.138	ketol-acid reductoisomerase, mitochondrial
FOXG-13625	2.501	0.021	MFS transporter, SP family, general alpha glucoside: H^+^ symporter	FOXG-07887	1.521	0.282	acyl-CoA dehydrogenase
FOXG-09382	1.09	0.559	vesicle transporter SEC22	FOXG-00735	2.385	0.051	acetyl-coenzyme A synthetase
FOXG-07958	3.395	0.015	PiT family inorganic phosphate transporter	FOXG-02925	1.63	0.209	acetyl-CoA acyltransferase
FOXG-05598	1.501	0.105	Ca^2+^: H^+^ antiporter	FOXG-11385	1.51	0.138	acetyl-CoA C-acetyltransferase
FOXG-07591	1.735	0.002	glutathione S-transferase	FOXG-06200	1.312	0.221	acetyl-CoA C-acetyltransferase
FOXG-05808	5.59	0.001	catalase	FOXG-05118	1.595	0.151	acyl-CoA dehydrogenase
FOXG-15530	3.23	0.001	peroxidase	FOXG-02255	5.651	0.001	endonuclease G, mitochondrial
FOXG-05132	2.871	0.023	oxidoreductase	FOXG-05827	−3.355	0.012	acetyl-CoA acyltransferase
FOXG-12260	1.525	0.253	catalase-peroxidase	FOXG-13050	−2.138	0.005	alcohol dehydrogenase

Note: Log_2_ (IA/CK) ≥ 1 indicates up-regulated expression and ≤−1 down-regulated expression.

**Table 3:** Differentially expressed genes in *F. oxysporum* exposed to 50 μg/mL iturin A compared with the control.

Gene ID	log_2_ (IA/CK)	*p*-Value	Annotated Gene Function	Gene ID	log_2_ (IA/CK)	*p*-Value	Annotated Gene Function
DOWN-REGULATED							
FOXG-19811	−5.152	0.035	tRNA	FOXG-05075	−1.38	0.005	60S ribosomal protein L27a
FOXG-00235	−1.817	0	DNA repair protein rhp51	FOXG-10571	−1.253	0.002	60S ribosomal protein L7
FOXG-02005	−1.615	0.002	ribosome biogenesis protein ytm-1	*Tri5*	−2.362	0.202	Synthetic enzyme gene
FOXG-10250	−1.383	0.001	ribosome biogenesis protein ERB1	FOXG-01535	−1.03	0.018	ABC transporter
FOXG-02122	−1.526	0.003	50S ribosomal protein S22	FOXG-07519	−1.252	0.005	mannitol-1-phosphate dehydrogenase
FOXG-01619	−1.327	0.006	50S ribosomal protein S29	FOXG-07382	−2.529	0.007	acid phosphatase
FOXG-03375	−1.253	0.007	50S ribosomal protein L15e	FOXG-10756	−1.73	0.002	acid phosphatase
FOXG-05325	−1.253	0.008	50S ribosomal protein L22e				
UP-REGULATED							
FOXG-11555	3.006	0.005	cytochrome P550, family 51 (sterol 15-demethylase)	FOXG-03076	1.015	0.039	superoxide dismutase
FOXG-08765	2.12	0.008	sterol 25-C-methyltransferase	FOXG-17180	1.537	0.006	catalase-peroxidase 2
FOXG-10530	1.151	0.005	C-5 sterol desaturase	FOXG-10652	1.651	0.005	S-(hydroxymethyl)glutathione dehydrogenase
FOXG-12390	1.135	0.027	MFS transporter, SP family, general alpha glucoside: H^+^ symporter	FOXG-08382	1.375	0.005	glutathione S-transferase
FOXG-10778	2.321	0.001	MFS transporter, SP family, general alpha glucoside: H^+^ symporter	FOXG-05237	1.373	0.011	glutathione S-transferase
FOXG-13625	2.682	0	MFS transporter, SP family, general alpha glucoside: H^+^ symporter	FOXG-13656	1.351	0.005	glutathione S-transferase
FOXG-15360	1.291	0.013	MFS transporter, SP family, sugar: H^+^ symporter	FOXG-07591	1.735	0.002	glutathione S-transferase
FOXG-17535	1.073	0.016	MFS transporter, SP family, sugar: H^+^ symporter	FOXG-01733	1.138	0.028	pyruvate carboxylase
FOXG-12267	1.252	0.008	MFS transporter, SP family, sugar: H^+^ symporter	FOXG-07887	1.521	0.282	acyl-CoA dehydrogenase
FOXG-13707	1.599	0.003	sodium/potassium-transporting ATPase subunit alpha	FOXG-00735	2.385	0.051	acetyl-coenzyme A synthetase
FOXG-05132	3.859	0.002	oxidoreductase	FOXG-11385	1.51	0.138	acetyl-CoA acyltransferase
FOXG-15750	3.637	0.011	oxidoreductase	FOXG-06200	1.312	0.221	acetyl-CoA acyltransferase
FOXG-00572	1.672	0.001	oxidoreductase	FOXG-08599	1.097	0.126	acetyl-CoA C-acetyltransferase
FOXG-09098	1.526	0.009	oxidoreductase	FOXG-05118	1.595	0.151	acyl-CoA dehydrogenase
FOXG-07588	1.585	0.01	oxidoreductase	FOXG-12010	2.312	0.035	hexokinase
FOXG-05703	1.055	0.026	oxidoreductase	FOXG-03031	1.238	0.02	hexokinase
FOXG-05808	6.563	0.001	catalase	FOXG-11536	1.577	0	acetoacetate-CoA ligase
FOXG-02357	1.779	0.02	catalase	FOXG-02255	5.758	0.02	endonuclease G, mitochondrial
FOXG-16836	1.565	0.02	catalase	FOXG-00035	1.521	0	enolase
FOXG-02505	1.036	0.059	catalase-1	FOXG-06150	1.281	0.003	6-phosphofructokinase
FOXG-15530	5.757	0.001	peroxidase	FOXG-02233	1.156	0.006	triosephosphate isomerase
FOXG-17180	2.558	0	peroxidase				

Note: Log_2_ (IA/CK) ≥ 1 indicates up-regulated expression and ≤−1 down-regulated expression.

## Data Availability

Data are contained within the article.
